# The most prevalent perceived barriers to sharing research data at the point of manuscript acceptance in health and life sciences: a survey at the University of Bristol

**DOI:** 10.12688/f1000research.161819.2

**Published:** 2025-08-15

**Authors:** Rrita Bajraktari, Fiona Booth, Marcus Munafo, Nicholas Beazley-Long

**Affiliations:** 1 Universite Libre de Bruxelles, Brussels, Brussels, Belgium; 2 University of Bristol Faculty of Life Sciences, Bristol, England, UK

**Keywords:** Health Sciences, Life Sciences, data sharing, open data, Open Science, reproducibility, reuse, barriers to sharing.

## Abstract

**Background:**

Sharing research data is critical for study validation and reuse, yet challenges persist across disciplines, such as psychology ^1^ and biomedical science ^2^. While global initiatives promote open science, understanding localized barriers in specific academic contexts is vital to implementing effective solutions.

**Objective:**

To investigate the most prevalent perceived barriers and reasons that prevent the sharing of research data underlying manuscripts, at the point of their acceptance for publication, within the Faculty of Health and Life Sciences (FHLS) at the University of Bristol, a research-intensive university in the UK.

**Methods:**

We distributed a comprehensive survey to FHLS researchers, addressing logistical, technical, and cultural challenges. A total of 143 participants provided insights into their experiences with data sharing.

**Results:**

The primary obstacles identified were time constraints and the complexity of the preparation process, with 34% reporting they “usually” or “always” lack sufficient time to adequately prepare their data for sharing. Additional barriers included not having the rights to share (27%), insufficient technical support (15%), and limited incentives within research teams. Moreover, qualitative responses highlighted a lack of confidence in data sharing infrastructure and guidance.

**Conclusions:**

These findings highlight the importance of targeted interventions to enhance data-sharing practices. Solutions should prioritize data preparation processes, clarify data ownership policies, and offer tailored training programs. Integrating data-sharing requirements into research workflows from the outset could significantly alleviate these challenges. Our study provides actionable recommendations to inform the development of resources and infrastructure that support a culture of open science within the FHLS at the University of Bristol.

## Introduction

Scientific research is inherently a collective enterprise. The era of solitary discoveries is long past; today, advancing knowledge requires collaboration, both within local research groups and across broader networks. Knowledge sharing has become essential to drive scientific progress and enhance our understanding through community efforts. For centuries, researchers have relied on publishing in peer-reviewed journals as the primary means of sharing their findings. However, accumulating evidence suggests that this traditional approach alone no longer meets the standards required by modern research for example in clinical trials ^3^ and societal challenges. ^4^ Poor replicability of research across disciplines in health and life science ^5^ underscores the need for greater openness and transparency in the research process. ^3^

Open research promotes openness beyond merely sharing final research findings. Key elements of openness include making datasets available when feasible and ethical, providing detailed documentation of methodologies, including metadata, and sharing research materials and code. Increased transparency not only allows for critical validation but also facilitates replication and enhances the credibility of both the research and researchers. ^6^

While data sharing was historically not standard practice, it is now an expectation, driven by shifts in research culture and mandates from funders and publishers. ^1^ However, transitioning to data sharing practices is far from straightforward, encountering challenges in definitions, ^7^ implementation, ^8^ ecological concerns (i.e. the energy needed to store the data being too high ^9^) and ensuring equitable practices. ^10^

For the purpose of this study, we define data sharing at the point of manuscript acceptance, as: the practice of making research data as open as possible online, allowing other researchers and the public to freely access, reuse, and analyse the data in a format which is openly available.

While the importance of data sharing is widely recognized, our study focuses on the practical and perceived barriers that researchers face when manuscripts are accepted for publication—a critical stage when data is finalized. By exploring these perceived barriers and reasons, we aim to help researchers recognize and overcome those challenges, where possible, to enable future data sharing. Our study highlights the main reasons for not sharing data within the Faculty of Health and Life Sciences (FHLS) at the University of Bristol, a research-intensive university in the UK, and reinforces findings from previous studies elsewhere, adding further evidence to support and expand on existing research and teaching in this area.

## Methods

### Study ethics approval

The survey design was approved by the University of Bristol’s School of Psychological Science Review Ethics Committee (Ethic approval: 18486, approved 05/06/2024). Informed consent was provided by each participant prior to beginning the survey, research data was anonymous at point of collection and no personally identifiable information was collected. The Participant Information Sheet and consent form are available with the other materials from this study.

### Survey design

To better understand the reasons and perceived barriers preventing the sharing of research data underlying manuscripts following their acceptance for publication, participants from across the Faculty of Health and Life Sciences were asked to rate statements related to data sharing. The survey consisted of two sections. In the first section, statements were framed as self-referential (e.g., “I don’t share data because it is too complicated”). In the second section, the same statements were rephrased to describe researchers’ behaviour around them (e.g., “They don’t share because it is too complicated”). The question was framed as “researchers around you”, to reduce social desirability bias – see survey statement development.

Participants were asked how often some barriers were encountered when sharing data at point of manuscript acceptance. They rated their responses using a four-point scale: “never,” “sometimes,” “usually,” and “every time.” For comparison, these responses were converted into numeric values ranging from 0 (never) to 3 (every time), and mean scores were compared across the two groups. Participants had the option to provide additional comments through a free-text box included at the end of each section. The survey was hosted on Microsoft Forms for online administration. Participants were asked to sign in using their university credentials to prevent entry duplication and external access by unauthorized individuals.

Participant instructions preceding the statements (see Extended Data for the full list of statements):

“On a scale from *Never* to *Every time*, can you rate how often each statement below **applies to you** when thinking about sharing the research data underlying your manuscript following its acceptance for publication:”

“On a scale from *Never* to *Every time*, can you rate on how often each statement below **applies to others around you** when thinking about sharing the research data underlying your manuscript following its acceptance for publication:”

### Survey advertising and incentive

The survey targeted health and life science researchers and was advertised through faculty research mailing lists and displayed on digital screens across the faculty. The survey remained open for one month. To encourage participation, three £50 prepaid gift cards were offered as incentives, with winners selected randomly using Google’s random number generator immediately following survey closure.

### Survey statement development

The survey items were developed based on themes from four key studies addressing data-sharing practices and barriers. Perrier and colleagues ^11^ identified issues such as data integrity, research conduct, and feasibility challenges, while Gomes et al. ^12^ emphasized barriers like process complexity, lack of incentives, and reuse concerns, and Gownaris et al. ^13^ highlighted early-career researchers’ concerns, including fear of misuse and career implications. Toelch and Ostwald ^14^ provided best practices for transparent research, shaping the phrasing of survey items.

A total of 21 items were selected from these themes. In the first section of the survey, the items were framed as personal statements (e.g., “I’m unsure about the process”) to foster self-reflection and participant engagement, consistent with findings from D’Ailly et al. ^15^ and Brenner. ^16^ The second section, where the statements applied to colleagues (e.g., “They are unsure about the process”), introduced psychological distance, which can reduce social desirability bias. ^17, 18^

### Thematic analysis of comments

After rating the statements, participants had the opportunity to leave comments, which were subjected to a thematic analysis. This analysis was conducted independently by two researchers using a systematic approach: read through all comments, then identify themes on second read-through, and categorize these themes into broader categories. Once both researchers completed their independent analyses, they shared and discussed their identified themes and categorizations to reach a consensus. In cases where disagreements persisted, a third researcher was consulted to resolve the differences and finalize the thematic categorization. This collaborative process helped to improve the reliability and validity of the thematic analysis. ^19^

## Results

### Participant demographics

A total of 143 active researchers completed the survey, composed of 114 research staff, 28 postgraduate research (PGR) students and 1 undergraduate student. The estimated response rate across the faculty research staff was 9.1% (Supplementary Figure S1) and researchers from all career stages contributed to the survey (Supplementary Figure S2).

### General survey findings

The primary perceived barrier to data sharing was a lack of time, identified as the top-ranked barrier in both sections of the survey. The statement “I/they don’t have enough time to prepare my/their data for sharing” had the highest mean scores in part 1 (personal framing: 1.19) and part 2 (colleague framing: 1.42).

**Figure 1.**
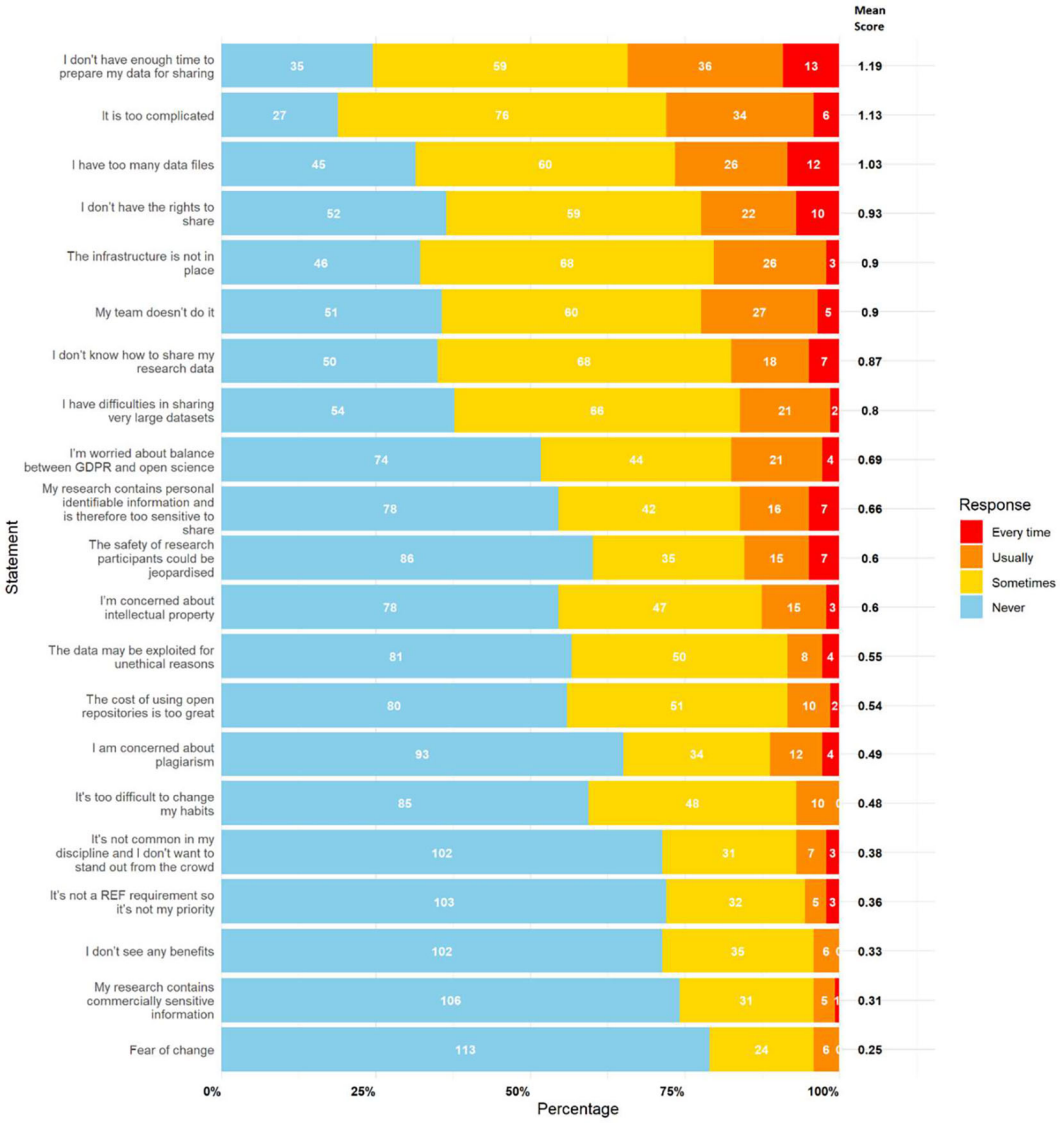
Results for perceived barriers to data sharing with personal framing. This figure illustrates participant responses to statements relating to perceived barriers to data sharing, framed as applying to themselves. The white numbers within each bar represent the absolute number of participants who selected “Never,” “Sometimes,” “Usually,” or “Every Time,” as indicated in the legend. The bold numbers on the right of each bar show the mean score for each statement. Mean scores were calculated by assigning numerical values to responses (0 = Never, 1 = Sometimes, 2 = Usually, 3 = Every Time), multiplying these values by the number of responses, and dividing the total by 143 (the number of participants).

**Figure 2.**
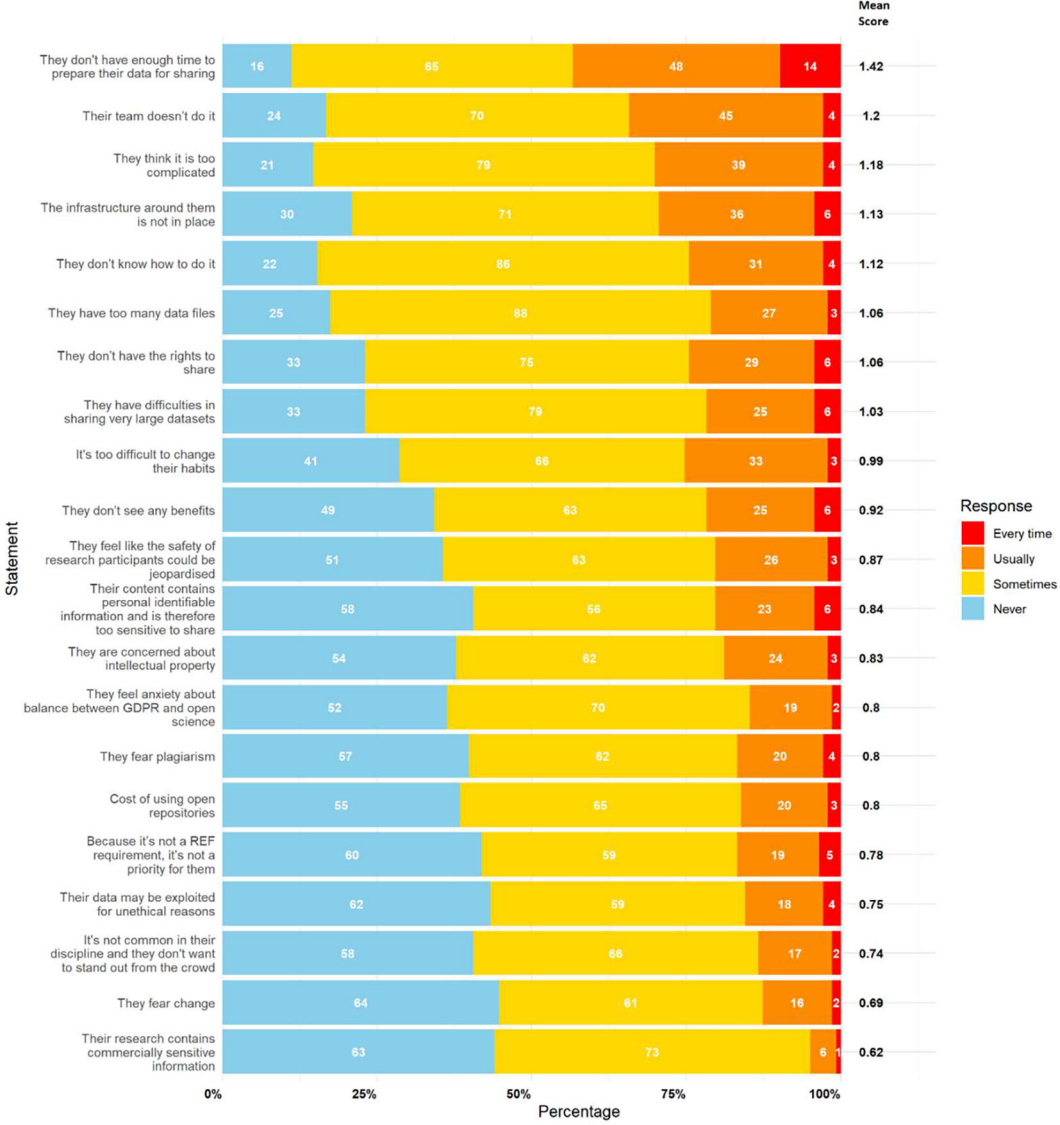
Results for perceived barriers to data sharing with colleagues framing (‘They’). This figure illustrates participant responses to statements relating to perceived barriers to data sharing, framed as applying to colleagues. The white numbers within each bar represent the absolute number of participants who selected “Never,” “Sometimes,” “Usually,” or “Every Time,” as indicated in the legend. The bold numbers on the right of each bar show the mean score for each statement. Mean scores were calculated by assigning numerical values to responses (0 = Never, 1 = Sometimes, 2 = Usually, 3 = Every Time), multiplying these values by the number of responses, and dividing the total by 143 (the number of participants).

Figure 1 (self-referential) and Figure 2 (colleague referential) rank the statements by mean score. The top ranked statements were:

• The lack of time to prepare data for sharing
• The complexity of the process (“too complicated”)
• Managing and sharing large datasets (“too many data files” and “difficulty sharing large datasets”)
• Lack of rights to share data
• Inadequate infrastructure
• Lack of team support (“my team doesn’t do it”)
• Lack of knowledge on how to share data

These results suggest that barriers are not only logistical but also cultural and knowledge-based. In part 2, similar barriers emerged but in a different order, with “Their team doesn’t do it” rising to second place. It is also interesting to observe that all mean scores for each statement were higher and that the top 8 statements in part 1 match those in part 2. This positive difference could be explained by a potential desirability bias or self-deception.

### Career stage and departmental differences

When breaking down the responses to the different career stages a similar general pattern to the combined responses was observed (Supplementary Figure S3), with “not enough time” being the top reason in two thirds of the career stages and the complexity around sharing (“too complicated”, “too many data files”) scoring highly. However, PGR students expressed more concerns about:

• Managing large datasets
• Risks of plagiarism
• Sharing intellectual property

Differences between departments (Supplementary Figure S4), aligning with other published findings, ^2^ also reflected disciplinary nuances:

• Population Health Sciences: The top barrier was the lack of rights to share sensitive data, reflecting the challenges and ethical concerns when handling health-related datasets.
• Psychological Sciences: Responses highlighted concerns about anonymising and sharing human-centred data.
• Biochemistry: The primary barrier was managing large and complex datasets.

These subtle differences between the schools and career stages will help us to tailor our internal training appropriately for difference audiences.

### Survey free text comments

Of the 143 participants, 57 left detailed comments, which were analysed thematically to provide additional insights. This process led to the identification of 14 themes consisting of some that were linked to the pre-defined statements (e.g. a lack of time), also others that were not covered by the statements (e.g. fear of failure to replicate, fear of error detection). This analysis has provided additional context enriching our understanding of the challenges and perceived barriers. The full thematic analysis is available in the available extended data. Below are examples of the identified themes and additional context.

• Data organization and clarity: disorganized datasets not designed for sharing can lead to misinterpretation.
• Training and support gaps: a lack of accessible guidance and resources on data sharing was frequently mentioned especially guidance on sharing qualitative data.
• Challenges in anonymizing qualitative data were highlighted by several participants.
• Fear of error detection and reproducibility issues: new concerns for our study emerged about criticism over errors or failed replication when data is shared.

Participants also highlighted distinctions within certain barriers, such as the “lack of benefit” theme. For example, 6 comments differentiated between a lack of personal benefit and a broader perception of the practice being “useless” for the field. This rich qualitative data will inform our training resources and workshops and enable the refinement of the statements for further iterations of the survey. The comments in full and the identified themes are available with the extended data. ^20^

## Discussion

This survey aligns with previous findings on the barriers to data sharing. ^11– 14^ For instance, the predominant challenges identified, such as time constraints, complexity of data sharing, and lack of incentives, mirror those reported in the literature. But while our results reveal consistent tendencies with these earlier studies, no significance tests were conducted.

Our findings highlight several areas that could be of some interest for further investigation. First, the analysis of participants’ comments suggests that greater specificity in survey items addressing complexity would be valuable. For example, the item “it is too complicated” could be refined to distinguish between technical challenges, organizational issues, and insufficient infrastructure. This distinction would enable future research to better identify and address specific barriers. Similarly, the item “I don’t see any benefits” should differentiate between personal perceptions (e.g., “no benefit for me”) and broader views of utility to the scientific enterprise. This level of detail could inform tailored interventions, such as workshops to raise awareness of data-sharing benefits or institutional policies offering incentives for researchers.

Another important consideration for future research is the impact of question phrasing on responses. Our survey’s dual framing of items (“I” vs. “They”) provides preliminary evidence of a potential social desirability bias. As data sharing becomes increasingly common, it is possible that open science practices are perceived as socially desirable behaviors, influencing self-reported attitudes and behaviors. Investigating this bias further could provide valuable insights into the evolving perception of open practices within the scientific community. To our knowledge, no prior survey has employed this dual framing approach, making our study a novel contribution to the evaluation of scientific practices.

Finally, the comments from our participants revealed an additional barrier not initially considered: fear of error detection or lack of reproducibility. This concern underscores the importance of fostering a culture of transparency that minimizes stigma around errors. An initiative to achieve this was recently launched with the *Estimating The Reliability & Robustness Of Research* (ERROR) project. ^21^ Future surveys should include items explicitly addressing this issue to capture its prevalence and impact on data-sharing behavior.

### Limitations and recommendations for future surveys

This study has several limitations that should be considered when interpreting the findings. First, participants were not given the option to skip statements they felt uncomfortable answering or did not know how to respond to, which may have affected the reliability of some responses. Second, we did not collect information about the primary research data type (e.g., quantitative or qualitative) for each participant. Including this information in future surveys could provide important context for understanding the specific barriers faced by researchers working with different types of data. Third, the sample size was limited for certain schools, which may have introduced bias in the representation of discipline-specific challenges. Conducting future surveys during periods when more researchers are available, such as outside of the summer months, could help improve response rates and ensure a more representative sample.

Despite these limitations, the survey has provided direct evidence of the barriers to data sharing at the University of Bristol and we consider these findings to be generalizable to other similar research-intensive institutions. These valuable insights have informed in-house training and guidance initiatives. Specifically, these results have shaped resources aimed at improving data management skills for data sharing and reproducible research practices. Additionally, this survey serves as a benchmark for future iterations, allowing for the evaluation of progress in addressing the barriers identified and refining strategies to support data sharing within the academic community.

## Ethical considerations and consent to participate

The survey design was approved by the University of Bristol’s School of Psychological Science Review Ethics Committee (Ethic approval: 18486, approved 05/06/2024). A Participation Information Sheet (PIS) was made available to the participants introducing the background and objectives of the study, that their research data (composed of their ratings to the statements and any comments) was anonymous at the point of collection, therefore not containing any personally identifiable information, and that the research data would be made open following the completion of the study. Written informed consent was collected digitally via Microsoft Forms before the survey began. Participants provided consent by ticking a checkbox. If consent was not given, the survey could not be started. Following the completion of the survey participants had the opportunity to enter a prize draw to win one of three £50 gift cards by submitting their university email address. These email addresses were stored separately, and not linked to, the anonymous research data and following the prize draw the email addresses were immediately deleted.

## Data Availability

Study data, code, figures and supplementary figures are openly available on the University of Bristol’s open repository ( https://doi.org/10.5523/bris.qldqe39bi5yd22hkg5q3zce3i) ^22^ Data and figures are available under the terms of the Creative Commons Attribution 4.0 International license (CC-BY 4.0) ( https://creativecommons.org/licenses/by/4.0/). The deposit includes the following:
• Readme.txt - A readme file including descriptive project metadata and the computing requirements to reuse the code •
UoB_FHLS_2024DataAvailabilitySurvey.csv – the raw data in csv • UoB FHLS 2024DataAvailabilitySurvey.xls – raw data in xlsx •
Excel_to_Figures_DATA_survey.R – code to generate figures This research followed the Consensus-based Checklist for Reporting of Survey Studies, CROSS. ^23^ The checklist accompanying this article is available. ^20^ Additional data and study materials are available in the project OSF deposit ^20^ ( https://doi.org/10.17605/OSF.IO/V2XP3) available under the terms of the Creative Commons Attribution 4.0 International license (CC-BY 4.0). This deposit includes the following:
• ParticipantInformationSheet.docx – the PIS • OnlineConsentStatementBarriersToDA.docx – consent statement • CrossSurveyGuidelines.docx – guidelines followed for this survey • F1000ResDAQualiThemeExtraction.xlxs – theme analysis of participants’ comments
