# Supplementary material for: The most prevalent perceived barriers to sharing research data at the point of manuscript acceptance in health and life sciences: a survey at the University of Bristol
Source: F1000Res. 2025 Aug 15;14:482. Originally published 2025 May 9. [Version 2] doi: 10.12688/f1000research.161819.2 (PMC12375187; doi:10.12688/f1000research.161819.2)
Supplement: Peer review history

# Reviewer report: Reviewer response for version 2

**DOI:** 10.5256/f1000research.186377.r407252

**Published:** 2025-10-10

**Authors:** Olavo B. Amaral, Gabriel G. Costa

**Affiliations:** 1 Federal University of Rio de Janeiro, Rio de Janeiro, Brazil

Summary:

 The study aims to investigate the most prevalent perceived barriers and underlying reasons that prevent the sharing of research data underpinning manuscripts at the point of acceptance for publication across Health and Life Sciences at the University of Bristol. The manuscript is clear and well-written, and the research question is relevant, although generalizability is limited by the specificity of the sample.

Major points:

 - There is a data inconsistency between the results section, which mentions 114 research staff responses and Supplementary Table S1, which mentions 115 responses. Please revise.

 - In spite of the statement “see Extended Data for the full list of statements”, we could not find the actual survey instrument in the OSF page, and could only find the statements in the raw data spreadsheet. Also, it would be much more useful to provide a direct link to such materials, rather than pointing to the Extended Data section, which then points to the general OSF page. The same holds for supplementary figures, which would be more easily accessible if they were made part of the paper (or via a direct link to each figure in the OSF).

 - We don’t think that the difference in mean scores between the “I” and “people around me” responses is evidence for social desirability bias. First, I’d assume that, due to responder bias, people answering such a survey will have above-average interest in data sharing and may be genuinely more prone to doing it than the those around them. Second, as the answer options are “never/sometimes/usually/always”, it is expected that the “never” and “always” categories should shrink if one is talking about a large group of people rather than about a single person (as variance in the larger group will lead “sometimes” and “usually” to be more likely). From the figure, this indeed seems to be the case. Reduction in “never” responses (which are the most prevalent responses in the personal section) would thus by itself lead to a greater average in the third-party condition, even with no bias present.

 - As the authors make some comparisons between response categories, it might be helpful to include 95% confidence intervals for mean scores to allow the reader to assess how likely these differences would be expected to happen by chance.

 - Given the low response rate, responder bias is an important issue here and could indicate skepticism about data sharing among non-responders. The potential consequences on the findings should be discussed explicitly in the limitations section.

 - Greater context about the University of Bristol is warranted if the authors want to imply that these findings are generalizable to other research-intensive institutions, so the reader can form an opinion about the scope of this generalizability. In terms of context, it would also be useful if the authors could discuss the implications of this data for potential policy changes in the institution.

Minor points

 - In the first paragraph, the phrase "Poor replicability of research across disciplines in health and life science" is supported by reference number five (5), which only addresses replicability in social psychology. It would be useful to include references on replicability in other areas as well.

 - In the second paragraph under survey design, it is written that "mean scores were compared across the two groups", but it is not clear which groups are being talked about (e.g. the groups of responses).

 - It is not clear from which schools the PGRs and the single undergraduate respondent came, and how they were contacted. Was the survey distributed exclusively via active research staff, or was it sent directly to PGRs and undergraduates as well?

 - It would also help to state more clearly in the methodology that the survey was conducted in the summer months, as this may have contributed to the low response rate

 - In the legends of Figure 1 and 2, the full meaning of GDPR should be stated.

Is the work clearly and accurately presented and does it cite the current literature?

Yes

If applicable, is the statistical analysis and its interpretation appropriate?

Not applicable

Are all the source data underlying the results available to ensure full reproducibility?

Yes

Is the study design appropriate and is the work technically sound?

Yes

Are the conclusions drawn adequately supported by the results?

Partly

Are sufficient details of methods and analysis provided to allow replication by others?

Yes

Reviewer Expertise:

Meta-research applied to research reproducibility

We confirm that we have read this submission and believe that we have an appropriate level of expertise to confirm that it is of an acceptable scientific standard, however we have significant reservations, as outlined above.

# Reviewer report: Reviewer response for version 2

**DOI:** 10.5256/f1000research.186377.r407248

**Published:** 2025-09-19

**Authors:** Anna Catharina Vieira Armond

**Affiliations:** 1 University of Ottawa Heart Institute, Ottawa, Canada

The study aims to investigate the barriers to research data sharing within the Faculty of Health and Life Sciences at the University of Bristol. The manuscript is clear and well-written, and the question is relevant. However, important clarifications and reporting gaps should be addressed.

 First, the manuscript would benefit from some clarifications about the survey development and administration:

- The full survey instrument is not included. I was only able to locate the survey statements by opening the dataset. The complete instrument, including instructions and questions, should be made available in the supplementary material or extended data.
- Briefly describing this process (or noting that it was not piloted) would be helpful.
- The timing of the survey is not specified (month and year), and it is not clear whether reminders were sent after the initial invitation. Adding this information would help readers interpret the reported response rates.

Results

- The authors report the response rate for staff, but not for the other groups. Response rates for all groups should be provided.
- Study participants are not fully characterized. While some demographics are reported in the main text, a more detailed description is needed, including career stage and department.
- Supplementary Figures 3 and 4 are important to the results and discussion. It would improve the paper if these were presented in the main text rather than as supplementary material.

Discussion and limitations

 Several points are missing or underdeveloped in the discussion. The study was conducted in a single faculty. While the results are useful for developing tailored interventions, the findings are not generalizable given the uneven representation across groups, the limited sample size, and the cultural specificity of data-sharing practices. This should be discussed with caution.

 The interpretation of the findings would also benefit from more institutional context. Data sharing is influenced by both cultural and institutional factors. Therefore, the authors may also wish to add more details about whether the University of Bristol has mandates or recommendations for data sharing, whether infrastructure, training, and resources are available, and whether local funders have relevant requirements.

 Finally, the definition of data sharing used in the study is restrictive, excluding controlled-access data, for example. This decision should be discussed, as biomedical researchers often face challenges in balancing de-identification with data usability, and controlled-access repositories are often the only viable model for sensitive health data.

Is the work clearly and accurately presented and does it cite the current literature?

Yes

If applicable, is the statistical analysis and its interpretation appropriate?

Not applicable

Are all the source data underlying the results available to ensure full reproducibility?

Partly

Is the study design appropriate and is the work technically sound?

Yes

Are the conclusions drawn adequately supported by the results?

Partly

Are sufficient details of methods and analysis provided to allow replication by others?

Partly

Reviewer Expertise:

Health sciences with a focus on metaresearch, research integrity, open science practices, and research data management.

I confirm that I have read this submission and believe that I have an appropriate level of expertise to confirm that it is of an acceptable scientific standard, however I have significant reservations, as outlined above.

# Reviewer report: Reviewer response for version 2

**DOI:** 10.5256/f1000research.186377.r405743

**Published:** 2025-08-23

**Authors:** Stefan Reichmann

**Affiliations:** 1 Graz University of Technology, Graz, Austria

Many thanks to the authors for carefully revising the paper. With the additional information on survey questions, the authors significantly improve the transparency of their methodology and the soundness of their argumentation. In the version that I reviewed, it was relatively unclear (at least to me) what questions had been used in they survey. Now that this has been appropriately addressed and included in the text, I will be happy to recommend the work for indexing.

Is the work clearly and accurately presented and does it cite the current literature?

Yes

If applicable, is the statistical analysis and its interpretation appropriate?

No

Are all the source data underlying the results available to ensure full reproducibility?

Yes

Is the study design appropriate and is the work technically sound?

No

Are the conclusions drawn adequately supported by the results?

No

Are sufficient details of methods and analysis provided to allow replication by others?

Yes

Reviewer Expertise:

NA

I confirm that I have read this submission and believe that I have an appropriate level of expertise to confirm that it is of an acceptable scientific standard.

# Reviewer report: Reviewer response for version 1

**DOI:** 10.5256/f1000research.177903.r386103

**Published:** 2025-06-16

**Authors:** Stefan Reichmann

**Affiliations:** 1 Graz University of Technology, Graz, Austria

Article: The perceived barriers to data sharing in health and life sciences: A survey at the University of Bristol

 Reviewer: Dr. Stefan Reichmann

 Article Synopsis

 The article investigates the perceived barriers to data sharing in the Faculty of Health and Life Sciences at the University of Bristol. A survey (n=143) was distributed addressing 3 types of perceived barriers: cultural, logistical, and technical. The survey found the primary obstacles to be time constraints and the complexity of the preparation process, with 34% reporting they “usually” or “always” lack sufficient time to adequately prepare their data for sharing. Additional barriers included not having the rights to share (27%), insufficient technical support (15%), and limited incentives within research teams. Moreover, qualitative responses highlighted a lack of confidence in data sharing infrastructure and guidance.

 Reviewer Comments

 This is a carefully executed study with a timely research question. Nevertheless, there are some problems with the work that should be addressed.

 The part of the work that I take issue with the most is the definition of data sharing given, which seems to me to be unnecessarily restrictive. The authors define data sharing as “the practice of making research data as open as possible online, allowing other researchers and the public to freely access, reuse, and analyze in a format which is openly available”. There are many other ways that data sharing happens, and by choosing such a restricted definition, the study risks missing quite a bit of the practice that may (or may not – this would be an interesting finding in its own right) be happening at FHLS. For instance, FHLS might have invested in data sharing infrastructure (an institutional repository, say), but respondents predominantly choose to share their data via other means or not share them at all – this would be undetectable with this survey. Christine Borgman (and others) have documented data sharing practices extensively, finding large variance across research settings in the ways research data are defined, produced, shared, reused, etc. I believe that defining data sharing as stated above restricts the domain of study too much – the phenomenon of data sharing is simply much broader than the study admits to. As is probably evident, this critique is written from the standpoint of a qualitative researcher who is mindful that the phenomenon of data sharing is richer and much more varied than the definition given.

 The narrowness of the definition has ramifications for the study design. Since the concept has not been defined vis-à-vis respondents (at least, this is not mentioned in the study), we do not know which definition of data sharing respondents subscribe to (whether a narrow definition, as is given by the authors, or a wide definition as I suggested above). In that sense, the responses given would be very difficult to interpret adequately.

 The scale used is not fit for the way the questions are phrased, at least in some cases (whenever the Likert scale is interpreted as “Never” to “Every time”): For example, “I don’t share data because it’s too complicated” seems to me to be a yes or no question. Applying the Likert scale in this (and other) cases seems forced, to say the least. In this particular example, a different question formulation (and scale) would have been more appropriate. In particular, the question combines two variables (“I don’t share data” and “Data sharing is complicated”) which make interpretation difficult and risks losing information. Additionally, it creates ambiguity in interpretation, because respondents could be assenting to either one of the two statements in their response. Because the question combines them, it becomes impossible to interpret meaningfully. A better way to approach this would have been to ask, in a separate question, whether respondents share their data, how often, with whom, and by what means, and then to ask about barriers.

 The gulf between the way the questions are phrased and the response options offered becomes even more apparent when considering the second framing of the questions (“they” instead of “I”). There, it seems that very few respondents chose the option “Every time” for all of the survey items. I suspect that this is due to the way the questions are phrased and does not necessarily reflect how respondents view their colleagues. At the very least, a considerable number of statements would have to be assessed based on an “Completely agree-Completely disagree”-Likert Scale. Also, many of the questions logically imply an “I don’t know”-option. That respondents did not have the option to skip questions either (a limitation the authors acknowledge), in my view, hampers the results further. This could have been solved relatively easily by including an “I don’t know/Cannot answer” option. In general, while a survey should be constructed so as to yield generalizable, quantifiable results, the questions should still be as close as possible to our normal way of speaking, i.e. even if responses are collected on a five-(four, three)-point-scale, the questions should be as close as possible to the way respondents normally speak about the issues in question. Consequently, either the statements or the response options need to be rephrased (where in fact, I would opt for the latter in most cases). In addition, I would need to see the questions that respondents were actually asked – at present, the article merely presents the statements that respondents rated but I suspect that there would have been some kind of prompt.

 Further, I am having trouble understanding the rationale of the two question framings. While I do think that reducing social desirability bias is laudable, I am hesitant to concur with the way this has been achieved because the resulting survey questions are difficult to interpret at face value – “they” is highly ambiguous, and the quality of a response changes considerably depending on how a respondent interprets “they” (colleagues can be people in the same department, field, even university – we don’t really know what group this is referring to). For instance, “They have difficulty sharing very large datasets” could be taken to refer to colleagues in the same field, which suggests a lack of skills, or it could be referring to colleagues in other disciplines, for which an explanation seems a little more complicated. In addition, some of the statements make considerably less sense when substituting “I” for “They”, for instance “Their team doesn’t do it” seems to me to be very difficult to interpret – whose team?

 In terms of the findings, I would expect them to mirror those found in the literature since the survey items have been developed based on landmark studies, but I would caution against interpreting these findings as simply confirming previous work; rather, because of the survey design, I would argue that the work reproduces the assumptions built into the work that the present article builds upon.

 In order to be of some help, I would like to end with some suggestions for how to improve the paper: The simplest (but probably most time-consuming) would be to follow-up with respondents across the disciplines represented in the sample and ask them for an interview. This would give respondents space to clarify how they interpret the core ideas used in the survey (such as “data sharing”, “reproducibility”, etc.), and to clarify whom they regard as in- resp. outgroup. Other than that, I think the most sensible course would be to revise the questions as indicated (I would be happy to offer my help there).

Is the work clearly and accurately presented and does it cite the current literature?

Yes

If applicable, is the statistical analysis and its interpretation appropriate?

No

Are all the source data underlying the results available to ensure full reproducibility?

Yes

Is the study design appropriate and is the work technically sound?

No

Are the conclusions drawn adequately supported by the results?

No

Are sufficient details of methods and analysis provided to allow replication by others?

Yes

Reviewer Expertise:

Sociology of Science, Science and Technology Studies, Open Science, Information Science

I confirm that I have read this submission and believe that I have an appropriate level of expertise to state that I do not consider it to be of an acceptable scientific standard, for reasons outlined above.

# Response

**Published:** 2025-08-08

**Authors:** Nicholas Beazley-Long

**Affiliations:** Psychology, University of Bristol, Bristol, Bristol, UK

We thank the reviewer for the constructive criticism. We have made several changes and additions to the article for clarity. We believe we have addressed all the reviewers concerns below. 

 Addressing the concern about the definition of data sharing used in this study. Our aim was to investigate the most prevalent perceived barriers and underlying reasons that prevent the sharing of research data underpinning research manuscripts, at the point of acceptance for publication across Health and Life Sciences at Bristol. To this end, we defined data sharing at the point of manuscript acceptance as: the practice of making research data as open as possible online, allowing other researchers and the public to freely access, reuse, and analyse the data in a format which is openly available. We have adjusted the text in the introduction to clarify this definition. To clarify that respondents were instructed to consider barriers to sharing data underlying manuscripts’ findings at the point of publication acceptance we have revised the title accordingly. We specifically chose this time point in the research lifecycle because at this point the data underlying manuscript findings are - in the vast majority - finalised and this time represents a critical decision-making moment when researchers are often required to consider data availability for publication. In addition, journal and funder policies around data sharing typically come into effect at this time. Focusing on this stage allows us to assess perceived and experienced barriers at the point when research data sharing is most actionable and policy-relevant. We have revised the article to include the text that instructed respondents how to rate the statements in part 1 and part 2. See below. These instructions help to define the ‘what’ (data underlying the manuscript) and the ‘when’ (at point of acceptance) for the respondents to consider. We did not want to state the exact types of data to consider (i.e raw, processed, summary, primary/secondary, methodological, unstructured etc) as this could have been restrictive. 

 The instructing text in the survey.

 Part 1. “On a scale from Never to Every time, can you rate on how often each statement below applies to you when thinking about sharing the research data underlying your manuscript following its acceptance for publication:” 

 Part 2. “On a scale from Never to Every time, can you rate on how often each statement below applies to others around you when thinking about sharing the research data underlying your manuscript following its acceptance for publication:” 

 Clarification of the Framing in Part 2 ("They" Framing) 

 The reviewer raised a concern that participants may not know what their colleagues do or don’t do in respect to sharing research data. However, the survey did not ask about colleagues specifically. The actual question posed was: “How often each statement below applies to others around you...?” This wording was intentionally chosen to capture participants’ perceptions of the data sharing practices and barriers surrounding them — not objective knowledge of others’ behaviours. This framing has been shown to reduce potential social desirability bias. 

 Clarification of the Question Format 

 Reviewer 2 also questioned the framing of some items, such as “I don’t share data because it’s too complicated,” suggesting these are yes/no questions. However, the survey asked participants to indicate how often each statement applied, not whether it applied or not. This frequency-based approach was deliberate: researchers may encounter certain barriers (e.g., complexity, time constraints) in some projects but not others. A binary yes/no format would have been overly restrictive and failed to capture this variability. By asking about frequency, we aimed to better understand the prevalence of different barriers across contexts. 

 Reproduction of assumptions argument 

 The reviewer makes the argument that our survey design reproduces assumptions built into the study on which this article builds. However, our intention was to assess the prevalence of barriers and underlying reasons for data sharing challenges in Health and Life Sciences research. To this end, we developed 21 statements based on existing literature, complemented by free-text boxes to allow respondents to share additional thoughts, including barriers not captured by the predefined items. This approach builds on established knowledge in the field, which we believe is essential rather than limiting and it would have been remiss of us to ignore. We acknowledge, that if our primary aim had been to uncover entirely new or undocumented barriers, a different methodological approach would have been needed. However, our focus was on identifying the most prevalent barriers in order to inform practical strategies for addressing them.

# Reviewer report: Reviewer response for version 1

**DOI:** 10.5256/f1000research.177903.r384382

**Published:** 2025-06-09

**Authors:** Juan-José Boté-Vericad

**Affiliations:** 1 Universitat de Barcelona, Barcelona, Catalonia, Spain

The article is well-organized and clearly communicates its objective.

 Current literature is cited across the introduction and methods sections.

 Some introductory sentences are slightly redundant or overly long. Introduction needs only minor stylistic refinements for clarity.

 Survey design is rigorous and includes ethical approval documentation.

 Dual framing (I/They) is a thoughtful method reducing bias.Thematic analysis methodology is clear, collaborative, and appropriately validated.

 Framing encourages reflection and distinguishes self versus group perceptions.

 Survey questions are described clearly and linked to past research.

 All source materials, survey documents, and code are openly available.

 Analysis steps are explained and data structure is well described.

 Qualitative coding steps include inter-rater review and consensus process.

 Raw data is shared via the University of Bristol repository.

 Files include survey results, R code, and metadata readme.

 Repository uses Creative Commons license ensuring open data access.

 Documentation enables re-use and verification by external researchers.

 No file or description appears to be missing or unclear.

 This article is suitable for publication after minor edits.

 Language in introduction and discussion could be slightly condensed.

 Consider clarifying broad terms like “too complicated” in future work.

 The study is robust, timely, and methodologically well executed.

 It contributes valuable evidence for improving data sharing culture.

 I recommend acceptance with very minor stylistic improvements.

 This is a useful model for open research infrastructure surveys.

Is the work clearly and accurately presented and does it cite the current literature?

Yes

If applicable, is the statistical analysis and its interpretation appropriate?

Not applicable

Are all the source data underlying the results available to ensure full reproducibility?

Yes

Is the study design appropriate and is the work technically sound?

Yes

Are the conclusions drawn adequately supported by the results?

Yes

Are sufficient details of methods and analysis provided to allow replication by others?

Yes

Reviewer Expertise:

Open Science

I confirm that I have read this submission and believe that I have an appropriate level of expertise to confirm that it is of an acceptable scientific standard.
